# Application of SNAP-Tag in Expansion Super-Resolution Microscopy Using DNA Oligostrands

**DOI:** 10.3389/fchem.2021.640519

**Published:** 2021-04-30

**Authors:** Longfang Yao, Li Zhang, Yiyan Fei, Liwen Chen, Lan Mi, Jiong Ma

**Affiliations:** ^1^Department of Optical Science and Engineering, Shanghai Engineering Research Center of Ultra-Precision Optical Manufacturing, Key Laboratory of Micro and Nano Photonic Structures (Ministry of Education), Green Photoelectron Platform, Fudan University, Shanghai, China; ^2^Shanghai Engineering Research Center of Industrial Microorganisms, The Multiscale Research Institute of Complex Systems, School of Life Sciences, Fudan University, Shanghai, China; ^3^Insititute of Biomedical Engineering and Technology, Academy for Engineering and Technology, Fudan University, Shanghai, China

**Keywords:** expansion super-resolution, DNA oligostrand, SNAP, F-actin, nuclear pore complex

## Abstract

Expansion super-resolution technology is a new technology developed in recent years. It anchors the dye on the hydrogel and the dye expands with the expansion of the hydrogel so that a super-resolution map can be obtained under an ordinary microscope. However, by labeling the target protein with a first antibody and secondary antibody, the distance between the fluorescent group and the actual target protein is greatly increased. Although fluorescent proteins can also be used for expansion super-resolution to reduce this effect, the fluorescent protein is often destroyed during sample preparation. To solve this problem, we developed a novel label system for expansion microscopy, based on a DNA oligostrand linked with a fluorescent dye, acrylamide group (linker), and benzoylguanine (BG, a small substrate molecule for SNAP-tag). This protocol greatly reduced the error between the position of fluorescent group and the actual target protein, and also reduced loss of the fluorescent group during sample preparation.

## Introduction

The optical microscope has been an important tool for biomedical research for a long time, because of its advantages of non-contact and no damage to the specimen. However, the diffraction limit, ~200–300 nm in the lateral direction, is comparable to or larger than many subcellular structures. Super-resolution fluorescence microscopy overcomes this limitation and provides an unprecedented tool for life science research (Huang et al., 2009). Stimulated emission depletion (STED) microscopy was the first super-resolution far-field optical microscopy technology; it utilizes a laser to suppress the fluorescence emission from the fluorophores located at the periphery of the excitation using stimulated emissions (Hell, 2015). STED microscopy can realize immunofluorescence imaging at 20 nm resolution and fluorescent protein imaging at 50–70 nm (Willig et al., 2006; Hein et al., 2008; Rankin et al., 2008). Single-molecule localization microscopy (SMLM) is based on the localization of individual fluorescent molecules. Typical examples of this kind of technology are photoactivated localization microscopy and stochastic optical reconstruction microscopy (STORM) (Betzig et al., 2006; Rust et al., 2006). Another method to improve the spatial resolution of optical microscope is structured illumination microscopy (SIM), which applies a patterned illumination field to the sample to obtain multiple images with illumination patterns of different phases and directions, thus reconstructing high-resolution images with a lateral resolution of ~100 nm and axial resolution of ~300 nm (Gustafsson, 2000; Gustafsson et al., 2008; Schermelleh et al., 2008). However, these three technologies have their own disadvantages. STED technology causes a large amount of light damage to the sample; SMLM technology has slow imaging speed and requires special fluorescent molecular markers; SIM technology has fast imaging speed, but lower resolution improvement.

In 2015, the Boyden group invented expansion super-resolution technology (Chen et al., 2015). This technology does not make changes to the microscope hardware but puts the fluorescently labeled biological sample in the swellable hydrogel so that the sample can be enlarged evenly as the polyacrylamide hydrogel swells. In this way, a super-resolution image can be obtained using ordinary microscope equipment. Combining expansion technology with traditional super-resolution optical imaging technology greatly increases the resolution, allowing observation of subtle structures and phenomena that were previously unobservable. For example, in 2018, expansion stimulated emission depletion microscopy (ExSTED), combined expansion microscopy and STED together, achieved super-resolution images with a resolution of <10 nm, which is 30 times higher than traditional microscopy techniques (Gao et al., 2018). In addition, the combination of expansion and STORM has also achieved good results. Using this technology in mouse spermatocytes achieved a resolution of 10–20 nm and showed a detailed structural view of the meiotic chromosome axis (Xu et al., 2019). The combination of extension and SIM can also increase the spatial resolution to 30 nm. Furthermore, ExFEAST, which combines fluctuation-enhanced Airyscan technology (FEAST) and sample expansion microscopy, improves the lateral resolution to ~26 nm (Wang et al., 2020). Its advantages of simple operation and fast but powerful functions provide great convenience for the research of fixed biological specimens and even more biological systems (Halpern et al., 2017).

At present, there are two main fluorescent labeling methods used in expansion super-resolution. One is to use a primary antibody to connect a fluorescently-coupled secondary antibody (Kunz et al., 2020). The other is to use a fluorescent protein fused to the target protein (Tillberg et al., 2016). After expansion, the fluorescence signal can be lost owing to various reasons. Under normal circumstances, the luminous efficiency, brightness, and anti-quenching ability of antibodies are much stronger than fluorescent proteins. Therefore, even if the fluorescent signal is lost during the preparation of the expanded sample, information from the sample can be obtained based on the remaining fluorescent signal. However, the disadvantage of antibody labeling is that their large size leads the luminescent group to be far away from the target protein. In addition, only a few kinds of antibodies are available, limiting their practicability. In non-expanded samples, this size of an antibody is ~20 nm, which does not cause a large error, but after expansion, the size of antibody can often reach more than 80 nm (Mikhaylova et al., 2015; Zwettler et al., 2020). Antibody size is a critical factor that limits further resolution improvement in expansion microscopy (Li et al., 2018). The advantage of labeling the target protein with fluorescent protein is that the distance error is small, but the loss of fluorescent protein during preparation of the expanded sample is relatively large. Therefore, significant information is often missing (Tillberg et al., 2016). To improve this situation, one method is to expand the sample first and then label. For example, a biotin-conjugated antibody is used to identify the target protein before expansion, and dye-labeled streptavidin is used to identify the biotin after expansion (Li et al., 2018). Compared to using antibodies before expansion, this method can reduce the distance between the luminescent group and the target protein and reduce the loss of fluorescent signal. However, additional methods are still needed to reduce the distance between the fluorescent dye and the target protein.

To solve this problem, we developed a novel labeling system suitable for expansion microscopy. The system is based on SNAP-tag and a DNA oligostrand. SNAP-tag, a small mutant of the DNA repair protein O6-alkylguanine-DNA alkyltransferase (20 kD, <5 nm), can be covalently modified using O6-benzylguanine substrates (BG) (Juillerat et al., 2003; Nieves et al., 2019). The DNA oligostrand that we designed is a double-stranded DNA of 20 bp modified with BG, Acrydite, and dye at both ends of the DNA oligostrand. Fluorescent dyes can be taken to the target protein through the connection between SNAP and BG, and Acrydite can participate in the polymerization process of hydrogels to reduce the loss of the fluorescent group. In addition, since the DNA oligostrand, still intact after proteolysis, is not enlarged with the hydrogel, we can achieve our goal to reduce the distance between the fluorescent group and the target protein after expansion. We applied this method to observe the structure of F-actin and the nuclear pore complex (NPC), and found that these structures could be seen more delicately, indicating that it has the potential for application in a variety of fields.

## Materials and Methods

### Materials

Vector pEGFP-N1 was used to create the plifeAct-SNAP-EGFP, plifeAct-SNAP, and pNup153-SNAP plasmids. After restriction digestion to generate an empty backbone, the appropriate fragments were inserted into pEGFP-N1.

### Modification and Synthesis of DNA Oligostrand

We designed the DNA single strands according to Chen et al. (2015) with some modification. The sequences of the single-stranded DNAs are as follows: 5′-Alexa647-GACGATGTATGCTTAGGGTCT-Acrydite-3′; 5′-Alexa488-GACGATGTATGCTTAGGGTCT-Acrydite-3′; and 5′-BG-GACCCTAAGCATACATCGTCTT-3′. The modified DNA single strands were synthesized by the Beijing Genomics Institution (Beijing, China). Using the annealing program of the thermocycler, the DNA oligostrands were formed by the polymerization of single-stranded DNA. DNA oligostrands synthesized from different single strands were named BG-dsDNA-Alexa647 and BG-dsDNA-Alexa488.

### Cell Culture

HeLa and U2OS cells were grown in DMEM containing 1 g/L glucose (Gibco/Invitrogen) supplemented with 10% fetal bovine serum and 100 U/mL penicillin-streptomycin in a 37°C tissue culture incubator. Before transfection, cells were cultured in chambered cover glasses overnight to 60–80% confluence. The cells were transfected with constructs for 24 h using Lipofectamine 3000 (ThermoFisher) according to the manufacturer's protocol. After transfection, cells were then seeded on a #1.0/18 mm diameter round cover glass (Menzel) for use.

### Labeling With DNA Oligostrand in Fixed Cells

For fixed cell labeling, cells expressing SNAP-tags were harvested at 60–80% confluence and rinsed twice with phosphate buffered saline (PBS) to remove dead cells and debris. After fixing with 4% paraformaldehyde (157-8, Electron Microscopy Sciences) and 0.1% glutaraldehyde for 15 min at 25°C, cells were permeabilized with PBS supplemented with 0.3% Triton-X100 for 3 min and rinsed with PBS three times. DNA oligostrands were incubated with fixed cells at a final concentration of 1 μM in PBS supplemented with 5% bovine serum albumin and 1 mM dl-dithiothreitol (DTT) for 2 h.

### Labeling of Live Cells With DNA Oligostrands

Coverslips covered in an ~60–80% confluent layer of HeLa and U2OS cells were transfected with related constructs, incubated in 37°C prewarmed 40 μg/mL digitonin (Cat#D141; Sigma Aldrich) in import buffer (20 mM Hepes, 110 mM KOAC, 5 mM NaOAC, 2 mM MgOAC, 1 mM EGTA, pH 7.3) for 15 s for F-actin and 3 min for NPC, and then rinsed twice with 1.5% polyvinylpyrrolidone (PVP) in import buffer. The cells were stained with DNA oligostrands at a final concentration of 1 μM in 1.5% polyvinylpyrrolidone supplemented with 1 mM DTT for 5 min. Cells were then fixed with 4% paraformaldehyde and 0.1% glutaraldehyde (A600875-0025, BBI Life Sciences, Shanghai, China) for 15 min at 25°C, rinsed with PBS, and mounted in ProLong Glass Antifade Mountant (P36980, Invitrogen, Thermo Fisher Scientific). In this study, for plifeact-SNAP-EGFP, DNA oligostrand BG-dsDNA-Alexa647 was used. For plifeAct-SNAP and pNup153-SNAP, DNA oligostrand BG-dsDNA-Alexa488 was used.

### Expansion Microscopy

Expansion microscopy was performed as previously described (Chozinski et al., 2016) with slight modifications. To form a gel, stained and fixed cells were incubated with a monomer solution containing 8.625% sodium acrylate (w/w) (408220, Sigma-Aldrich), 2.5% acrylamide (w/w) (A4058, Sigma-Aldrich), 0.15% *N, N*, -methylenebisacrylamide (w/w) (M7279, Sigma-Aldrich), 2 M NaCl (S5886, Sigma), and 1 × PBS at room temperature and supplemented with fresh 0.2% (w/w) ammonium persulfate (APS, 17874, Thermo Fisher Scientific, Waltham, MA) and 0.2% (w/w) tetramethylethylenediamine (TEMED, 17919, Thermo Fisher Scientific, Waltham, MA) when used. Polymerization was performed for 2 h. The diameter of the gelated samples was measured to calculate the expansion ratio. A total of 8 U/mL proteinase K (EO0491, Thermo Fisher Scientific, Waltham, MA) in digestion buffer (50 mM Tris pH 8.0, 1 mM EDTA, 0.5% Triton X-100, 0.8 M guanidine HCl) was added to the gelated samples to digest cells. The digested cells were then expanded in ddH_2_O, and the water was changed every 30 min until the gel size no longer changed. The diameter of the gel was measured to calculate the expansion factor with dimensions before expansion. The expanded gels were stored in ddH_2_O at 4°C until use. For imaging, the gel was fixed with 3% low melting point agarose near the corner of the gel.

### Imaging

Unexpanded and expanded specimens in 35 mm glass bottom dishes were fixed as described in the previous protocol. Wide-field fluorescent images were collected onto an EMCCD camera (Evolve 512, Delta Photometrics) with an oil-immersed objective (150 × /1.45, Olympus), yielding a pixel size of 106 nm. Emissions were collected through an objective and filtered by a 520 nm band-pass filter (FF01-520/35-25, Semrock) for Alexa488 or by a 655 nm band-pass filter (FF02-655/40-25, Semrock) for Alexa647. Airyscan imaging was performed using a commercial microscope (ZEISS, LSM880, Germany) with an additional Airy FAST detector module (Zeiss), equipped with a Plan-Apochromat 63 × /1.4NA oil objective (Zeiss; Plan-Apochromat 63 × /1.4 Oil DIC M27). Samples stained with BG-dsDNA-Alexa488 were excited using a 488 nm laser. Emissions were collected through a 495–550 nm band-pass filter, 570 nm long-pass filter, and a 1.25 airy unit (~60 μm) pinhole onto a 32 GaAsP detector element. Pictures were obtained using ZEN software (Zeiss; black edition) and pixel size was set to ~40 nm in the x and y directions. SIM imaging was performed using a commercial microscope (NIKON, A1&SIM&STORM, Japan) with a SIM model equipped with a 100 × /1.49NA oil objective (Nikon; CFI Apo TIRF 100 × /1.49 Oil), and the 488 laser was selected.

### Image Processing and Analysis

Airyscan images were processed using ZENs Airyscan processing with automatic deconvolution parameters. Fluorescence images were analyzed using ImageJ and Origin software. A total of 300 images obtained by Airyscan mode were used to achieve reconstruction using the NanoJ-SRRF plug in ImageJ FEAST image (Gustafsson et al., 2016; Wang et al., 2020). SIM reconstruction was automatically constructed by Nikon software. According to the size changes before and after the expansion of the hydrogel, we determined that the expansion coefficient was 4.10 ± 0.05.

## Results

### Design of DNA Oligostrands

**Figure 1:**
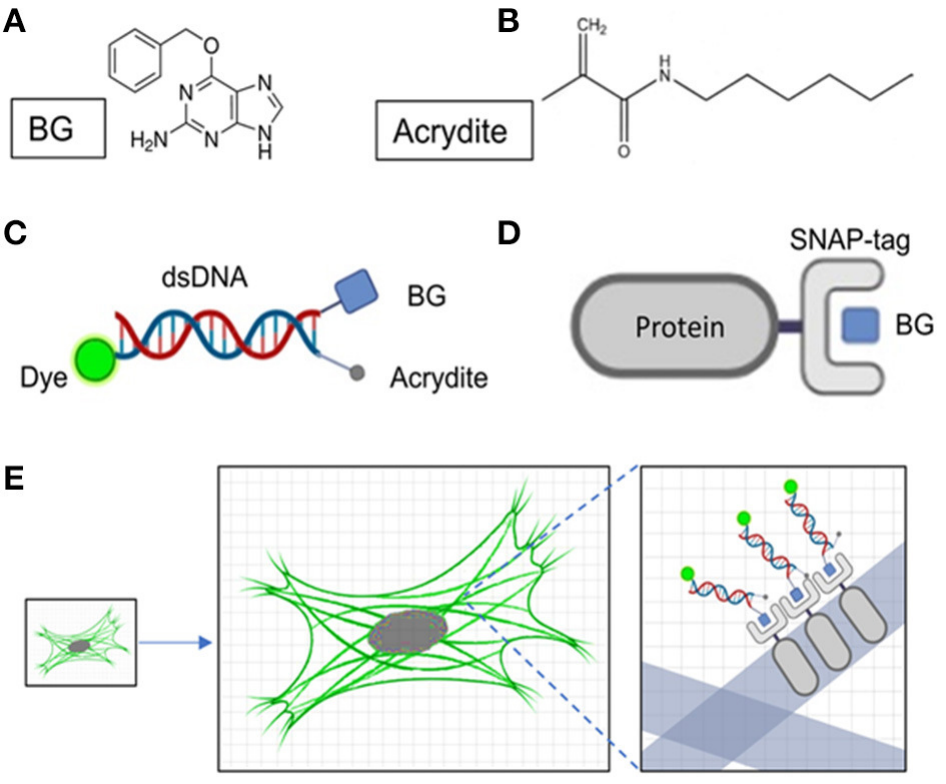
Design of DNA oligostrands and schematic diagram of SNAP-benzoylguanine (BG) staining. **(A)** Molecular formula of BG. **(B)** Molecular formula of Acrydite. **(C)** Schematic diagram of the designed DNA oligostrands. **(D)** Schematic diagram of SNAP-tag protein binding to BG. **(E)** Staining and expansion of the DNA oligostrand.

The BG molecule is a ligand of the SNAP protein; they can combine with each other in the cell to form a stable covalent bond (Figures 1A,B,D). To reduce the distance between the fluorescent group and the target protein, we designed DNA oligostrands of 20 bp. It is not only small in size (~6.8 nm), but also not enlarged as the hydrogel enlarges, for it cannot be degraded by proteinase K. The main body is a double-stranded DNA, modified with a BG molecule, fluorescent group, and Acrydite at the ends (Figure 1C). For the target protein to be labeled with the designed DNA oligostrand, we needed to express the target protein by fusion with SNAP, a small tag that can also reduce the distance between the target protein and the fluorescent signal after expansion to a certain degree. Through binding between BG and SNAP, the DNA oligostrand chain can bring the fluorescent group to the vicinity of the target protein. Acrydite connects the sample to the gel by participating in the polymerization process of hydrogels. DNA oligostrands can be designed with different fluorescent groups according to different experimental requirements. To perform experiments, we only need to transfect the relevant plasmid and wait for it to be expressed in the cell, and then incubate with the designed DNA oligostrands to stain the cell. Next, by following the protocol of sample expansion, we can achieve amplification of the target protein in the cell (Figure 1E). For this research, Alexa647 and Alexa488 were utilized. Alexa647 was chosen to distinguish from EGFP-labeled F-actin. Alexa488 was chosen for subsequent expansion super-resolution technology because it retains more signal after expansion than Alexa647 (Tillberg et al., 2016).

### Cell Staining After Fixation

**Figure 2:**
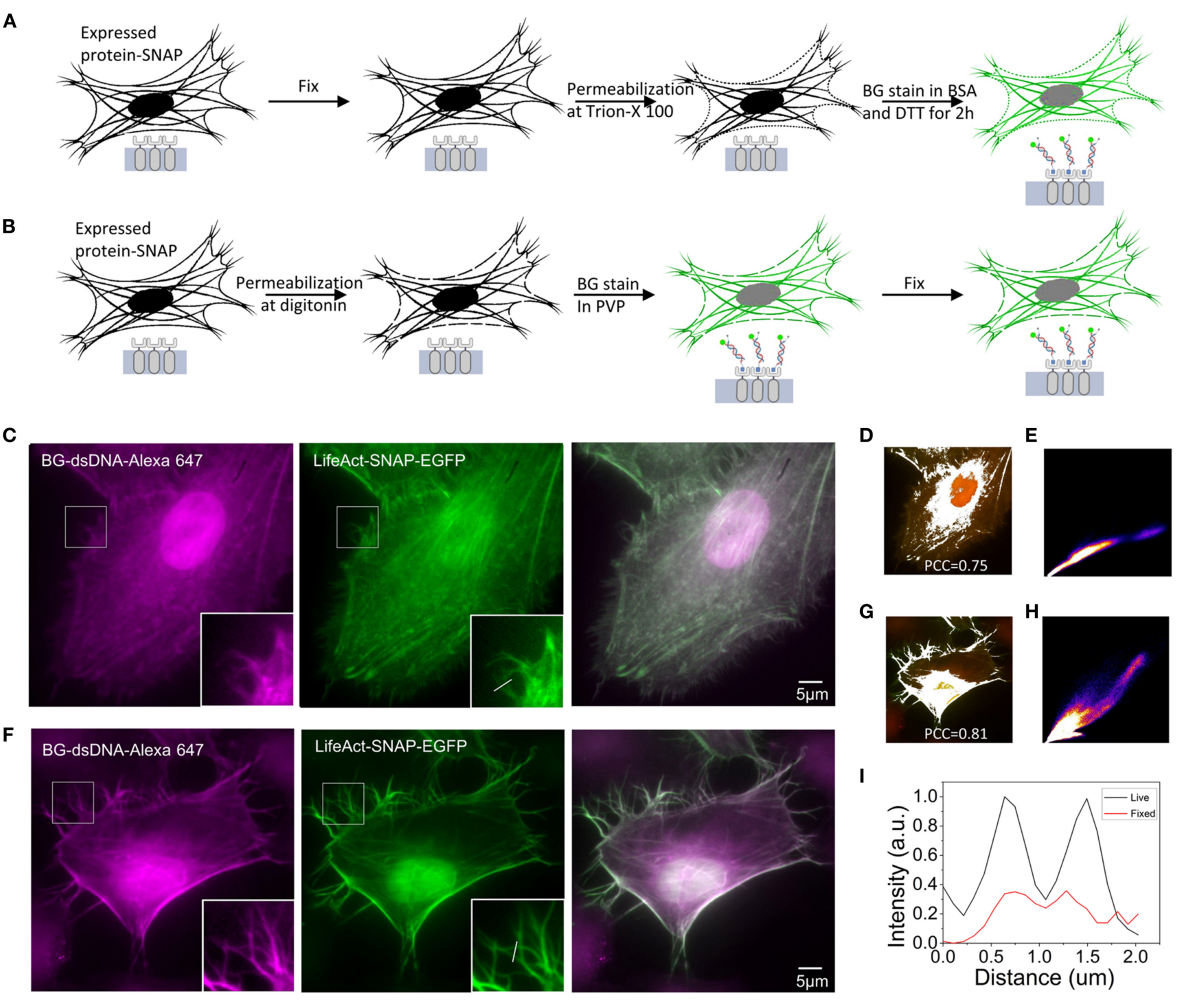
Comparison of F-actin staining using two methods in Hela **(A)** Schematic diagram of the process of staining after cell fixation. **(B)** Schematic diagram of the process of staining and then fixing cells. **(C)** Staining of F-actin with benzoylguanine (BG)-dsDNA-Alexa647 after fixation. **(D)** Simulation effect diagram of the co-location of the two channels in **(C)**. The Pearson correlation coefficient (PCC) is 0.75. **(E)** Distribution of the correlation scatter plot of the two channels in **(C)**. **(F)** Staining of F-actin with BG-dsDNA-Alexa647 before fixation. **(G)** Simulation effect diagram of the co-location of the two channels in **(F)** The Pearson correlation coefficient (PCC) is 0.81. **(H)** Distribution of the correlation scatter plot of the two channels in **(F)**. **(I)** Corresponding line-scanning profile from the images **(C,F)**. Scale bars, 5 μm.

Since the DNA oligostrand is essentially a piece of double-stranded DNA, it cannot directly penetrate the cell membrane and bind to the SNAP-tag protein expressed inside the cell, thus, making it impossible to directly stain the target protein. Regarding how to use the DNA oligostrand to stain the SNAP-labeled target protein, we devised a fixed cell staining method described below (Nieves et al., 2019). First, cells were incubated with 4% paraformaldehyde for 15 min, then permeabilized with Triton X-100 and stained with dye (Figure 2A). We first used the plifeact-SNAP -EGFP plasmid to transfect cells at a confluence between 60 and 80% and incubated them for 24 h to allow expression of the fusion protein. Next, we used the designed DNA oligostrand with Alexa647 to stain F-actin in the cells. At the same time, we used genetic engineering methods to label F-actin with EGFP to help verify the accuracy of the DNA dye-labeled site. From the light emitted by the DNA oligostrand and EGFP, we observed that the light-emitting areas of the two overlapped strongly and both stained F-actin (Figure 2C). Figure 2D is a simulation effect diagram of two channel co-localization, and the Pearson correlation coefficient (PCC) was 0.75. The distribution of the correlation scatter plot of the two channels is shown in Figure 2E. The staining results showed that the DNA oligostrand stained F-actin in the cell after cell fixation. However, the status of F-actin in the whole cell was not good, and the pseudopodia of the cell was not clear (Figure 2C). The results showed that a large number of microfilaments were depolymerized during the entire sample preparation process, and the cell morphology could not be well-maintained. Although this dyeing method can label F-actin, the result is not satisfactory. For this reason, we explored a new method.

### Optimization of the Staining Method

The new dyeing method is based on the properties of PVP, which can prevent osmotic swelling of cells. The method involves staining the cells while the cells are alive before fixation. Figure 2B shows the operation parameters. We first treated the cells with digitonin for 15 s to form small holes in the cell membrane, quickly incubated the cells with the DNA oligostrand in PVP for a short time, and then fixed the cells. Pore damage caused by digitonin to the cells is minimal, and cells are always in an environment with PVP; therefore, the cells remain alive during the short processing time (Ma and Yang, 2010). Since BG binds to SNAP very quickly, only a short incubation is sufficient to stain the cells. The stain results are shown in Figure 2F. With this new method, F-actin in the cells was stained and the cell morphology was relatively complete. The details were well-preserved, and the pseudopodia of the cells were clearly visible (Figure 2F). The PCC of the two channels was 0.81(Figure 2G), and its correlation was also greater than the stained image after fixation. The distribution of the correlation scatter plot of the two channels is shown in Figure 2H. The corresponding line-scanning profile from the images (Figures 2C,F) shows that the signal-to-noise ratio of the improved dyeing method is better than the previous dyeing method (Figure 2I). Compared with the staining method after fixation, now the F-actin structure of cells was better preserved.

### Expansion Super-Resolution

**Figure 3:**
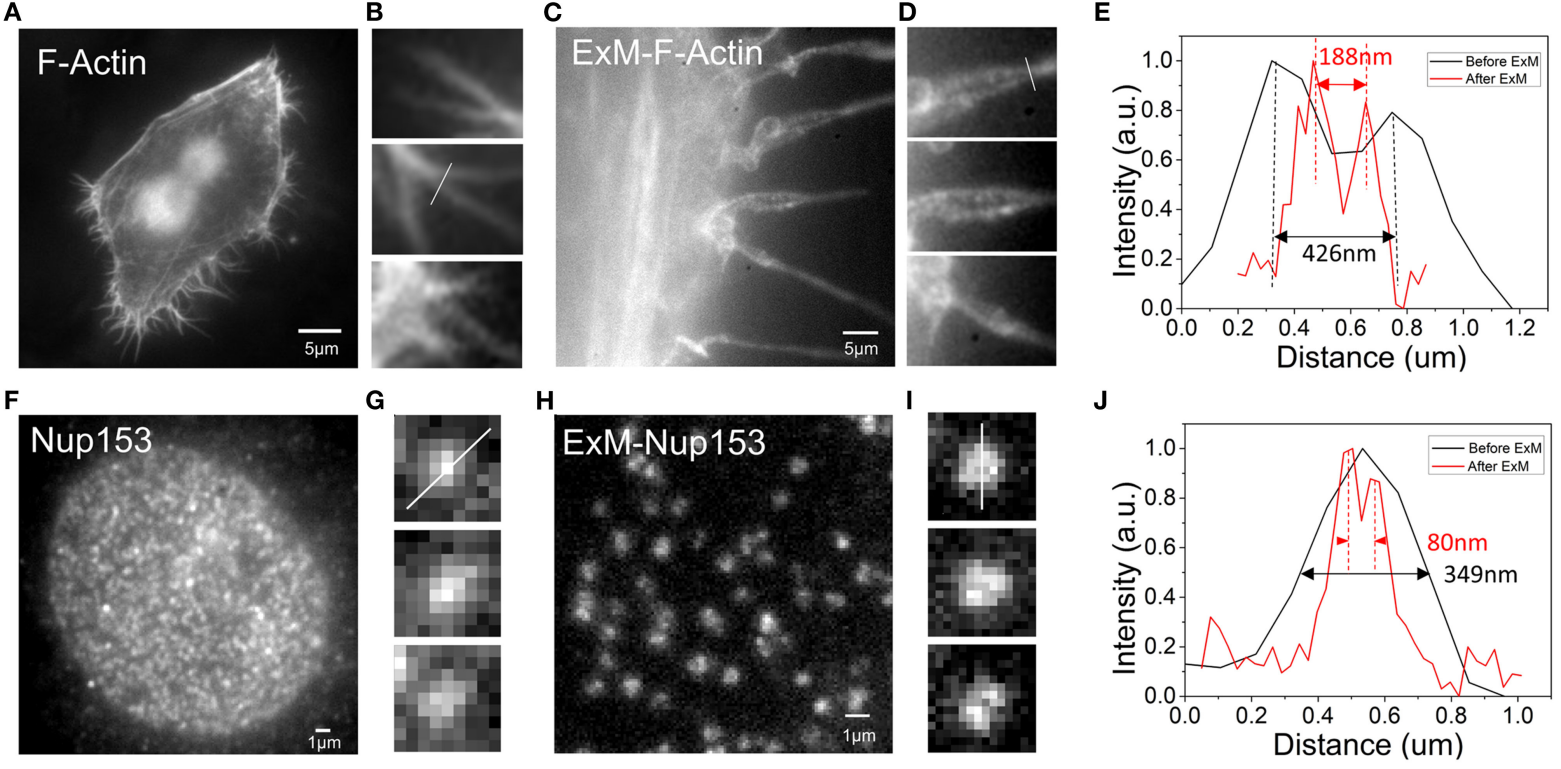
Comparison of the results of F-actin in Hela and NPC in U2OS before and after expansion **(A)** F-actin stained with benzoylguanine (BG)-dsDNA-Alexa488 before expansion. **(B)** Enlargement of part of picture **(A)**. **(C)** F-actin stained with BG-dsDNA-Alexa488 after expansion. **(D)** Enlargement of part of picture **(C)**. **(E)** Intensity distribution plot along arrows in **(B,D)**. **(F)** nNup153 stained with BG-dsDNA-Alexa488 before expansion. **(G)** Zoomed in view of **(F)**. **(H)** nNup153 stained with BG-dsDNA-Alexa488 after expansion. **(I)** Zoomed in views of **(H)**. **(J)** Line profiles of selected nuclear pore complexes in **(G,I)**. Scale bar: 5 μm in **(A,C)**, 1 μm in **(F,H)**.

To observe whether our method was suitable for expansion super-resolution, we performed expansion experiments after dyeing. For F-actin, more pseudopod details were observed after expansion. Comparing the results before and after expansion, we found that more pseudopods were formed by the aggregation of several bundles of fiber filaments (Figures 3A,C). Figures 3B,D are enlarged details of Figures 3A,C. Fitting the cross-sectional (Figure 3B) section of F-actin before expansion to a Gaussian peak, we determined the full width at half maximum (FWHM) to be 426 nm (Figure 3E). Similarly, we determined the FWHM in Figure 3D after rescaling by the expansion ratio after expansion to be 188 nm, which is smaller than pre-expansion (Figure 3E). The resolution barrier inherent in light microscopy restricts the ability to differentiate between objects closer than 300 nm (Thorley et al., 2014). However, after expansion, we could observe structures with a bifurcation of <300 nm in the pseudopodia under a wide field of view. The results obtained in the experiment show that the improved resolution after expansion allowed us to see more of the local structure of F-actin. To further test its application in analysis of different cellular structures, we next performed imaging of the NPC, which has great challenges for high-resolution structural research (Grossman et al., 2012). In our experiment, we imaged the organization of Nup153, a stable component of the NPC (Winterflood and Ewers, 2014). According to the results shown in Figure 3F, the nuclear pores before expansion were densely distributed, but were relatively sparsely distributed after expansion (Figure 3H). Fitting the cross-sectional (Figures 3G,I) section of the NPC before and after expansion to a Gaussian peak, we obtained the FWHM after rescaling by the expansion ratio after expansion to be 80 nm, which is smaller than the 349 nm determined before expansion (Figure 3J). Note that the image scale bars for expanded specimens in this report have not been divided by their respective measured expansion factors of 4.10 ± 0.05, but the data processing distances refer to pre-expansion dimensions.

### Analysis of the NPC

**Figure 4:**
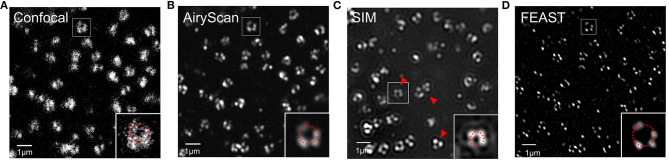
Combination of expansion super-resolution and other super-resolution technologies **(A)** nNup153 taken in confocal mode after expansion. **(B)** nNup153 taken in Airyscan mode after expansion. **(C)** Image of nNup153 after structured illumination microscopy reconstruction. **(D)** Image of nNup153 after fluctuation-enhanced Airyscan technology reconstruction. Scale bars, 1 μm.

Combining expansion super-resolution technology with other super-resolution technologies enabled us to further analyze the structure of the NPC. For the same field of view, we used confocal and Airyscan modes to photograph and SRRF to process the images taken by Airyscan to obtain a FEAST image. We also used SIM mode to photograph and reconstruct the NPC. In the picture taken in confocal mode, we found that the hollow structures of most NPCs were faintly visible (Figure 4A). In the picture taken in Airyscan mode, the hollow structure of most NPCs was clearer than in the confocal picture (Figure 4B). In the SIM mode, we also observed the hollow structure of the NPC (Figure 4C). However, we found some arc artifacts in the SIM reconstruction image (red arrow in Figure 4C) but not in other images. The FEAST image was the clearest (Figure 4D). The resolution of the NPC image processed by FEAST was higher than that of the other three. In addition, based on the statistics of 100 nuclear hole diameters processed by FEAST, the average diameter of the Nup153 ring was ~102 nm (Supplementary Figure 1), comparable to a previously reported measurement of Nup153 (Winterflood and Ewers, 2014). It is also comparable to that of other NPC components, such as Nup96 (Szymborska et al., 2013) and Nup133 (Li et al., 2018).

## Discussion

Expansion super-resolution technology has become popular tin recent years. Because of its simple operation and low instrument requirements, it can be quickly applied to various fields (Düring et al., 2019; Gambarotto et al., 2019; Götz et al., 2020). However, the shortcomings of expansion super-resolution technology are slowly being identified. Part of the signal can be lost during the formation of the hydrogel, and as the hydrogel expands, the distance error caused by commonly used antibody markers is amplified by the same multiple (Li et al., 2018; Zwettler et al., 2020). Therefore, a method with less signal loss and a small size is needed. We designed a new 20 bp DNA oligostrand BG-dsDNA-dye based on the characteristics of high specific binding between BG and SNAP and their small size. We used the DNA oligostrand to stain living cells, which is suitable for expansion super-resolution technology. Acrydite connected to the BG-dsDNA-dye can directly participate in the formation process of the hydrogel, thus reducing signal loss during the expansion process. Although other three functional linkers have been reported, this linker is very suitable for small molecule targeting of fluorophores (Wen et al., 2020). However, only some of the structures could be labeled well with small-molecules or antibodies. Our three function linker with genetic SNAP tag should be applicable for most proteins. The size of SNAP and BG-dsDNA-dye is also smaller than the size of most antibodies; therefore, the distance error between the fluorescent signal and the target protein can be reduced. A wider range of proteins than antibodies can be labeled by genetic engineering. Only a small number of proteins have their mature antibodies. Many antibodies would cause complex backgrounds as their unspecific binding problem. Using this method combined with expansion super-resolution, we have obtained a more detailed structure of F-actin and the NPC. The results show that the designed DNA oligostrands are suitable for expansion super-resolution technology and have wide applications.

Current super-resolution microscopy technology has improved the range of optical resolution from ~250 to ~10 nm, providing us with a powerful tool for studying the details of matter (Galbraith and Galbraith, 2011). When using antibodies to label the target protein, the resolution can be accurate to tens of nanometers. However, with the improvement of resolution, the influence of errors caused by the size of the antibody itself also increases. The accuracy of the location of the target protein is also greatly reduced. In expansion super-resolution technology, errors caused by the size of the antibody owing to swelling of the hydrogel are also amplified, which can be as high as 80 nm (Zwettler et al., 2020). Therefore, it is necessary to develop new labeling technology to reduce the labeling errors. Labeling after expansion is a way to reduce labeling errors since the antibody will not be enlarged with the enlargement of the hydrogel (Li et al., 2018; Zwettler et al., 2020). In addition, labeling errors can be directly reduced by reducing the size of the marker. In our method, the distance between the luminescent group and the target protein is determined by the size of the SNAP and the length of the DNA oligostrand. We obtained a reduction ratio of 1.09 through the relative molecular mass ratio of EGFP (26 kD) and SNAP (20 kD) (Deng et al., 2011). By referring to the size of EGFP (3.5 nm), we obtained an estimated SNAP diameter of 3.2 nm based on the reduction ratio. A 20 bp DNA double strand can form approximately two helices with an average pitch of 3.4 nm, so the size of the DNA oligostrand size is ~6.8 nm. The orientation between SNAP and DNA oligostrand is uncertain. When the relationship between SNAP and DNA oligostrand is vertical, the distance between the target protein and the luminescent group is the shortest. When the relationship between SNAP and DNA oligostrand is parallel, the distance between the target protein and the luminescent group is the longest. Therefore, the distance between the target protein and the luminescent group before expansion is in the range of 7.5–10 nm. After expansion, the size of SNAP is stretched four times to as large as 12.8 nm owing to digestion with proteinase K. The size of the DNA oligostrand remains unchanged at 6.8 nm because the DNA oligostrand is not digested by proteinase K. Therefore, the distance between the target protein and the luminescent group after expansion is in the range of 14.5–19.6 nm. Before or after expansion, the distance error caused by labeling in this study is smaller than the error in conventional antibody labeling. Therefore, the method in this study is not only suitable for expansion super-resolution technology, but also has fewer labeling errors.

Although our method labels F-actin and NPC well, the labeling efficiency we found was not as remarkable as expected according to the results of the NPC. NPC is a macromolecular assembly of eight-fold symmetrical arrangement of protein subcomplexes (Grossman et al., 2012). The pictures revealed that not all Nup153 molecules could be equally recognized by the marker. This may be due to the existence of endogenous Nup153 which causes not all Nup153 could be recognized by DNA oligostrands. Further work should be carried out to increase the labeling efficiency. As far as the scope of application is concerned, our method is suitably applied to the study of the subcellular structure, while not for the research of rapid cell activities. For the time-consuming activities that cell can be fixed in each state, our method still has reference significance. In addition, it is uncertain whether the results obtained in this study remain the same when the experimental conditions such as cell types or growth conditions are changed. However, it is still a good marking method to reduce errors and signal loss in expansion super-resolution technology. It helps to improve the localization accuracy of expanded samples and provides a technical basis for subsequent studies of the expansion super-resolution technology. The strategies developed in this work can also be used to combine expansion super-resolution microscopy with other imaging techniques.

## Conclusions

In summary, since the advent of the expansion super-resolution technology in 2015, it has been widely used in biology and other fields because of its simple operation and low equipment requirements. However, because the commonly used antibody labeling technology and fluorescent protein labeling technology have shortcomings, such as large size of the marker after expansion or loss of signal when used in expansion super-resolution technology. We designed a DNA oligostrand, and connected BG, Acrydite, and a fluorescent dye to the ends of the oligostrand. The DNA oligostrand is brought to the vicinity of the target protein by specific binding between BG and SNAP. Expansion super-resolution microscopy in combination with other super-resolution techniques showed that the DNA oligostrands are suitable for expansion super-resolution technology. The use of oligostrands in expansion super-resolution also achieved a better resolution of the structures of F-actin and the NPC. Compared with antibodies, DNA oligostrands have several advantages, including simple operation, suitability for expansion, smaller distance errors, and wide application feasibility.

## Data Availability Statement

The original contributions presented in the study are included in the article/Supplementary Material, further inquiries can be directed to the corresponding authors.

## Author Contributions

LY and JM conceived the project. LY designed the experiments and performed the imaging processing and data analysis. LY and LZ completed the experiment together and wrote the manuscript. LY, LZ, LM, and JM participated in revising the manuscript. All authors contributed to the article and approved the submitted version.

## Conflict of Interest

The authors declare that the research was conducted in the absence of any commercial or financial relationships that could be construed as a potential conflict of interest.

## Supplementary Material

Supplementary Figure 1 Diameters of Nup153 ring measured in FEAST images.
