# Supplementary figures and images for: Application of SNAP-Tag in Expansion Super-Resolution Microscopy Using DNA Oligostrands
Source: Front Chem. 2021 Apr 30;9:640519. doi: 10.3389/fchem.2021.640519 (PMC8119759; doi:10.3389/fchem.2021.640519)

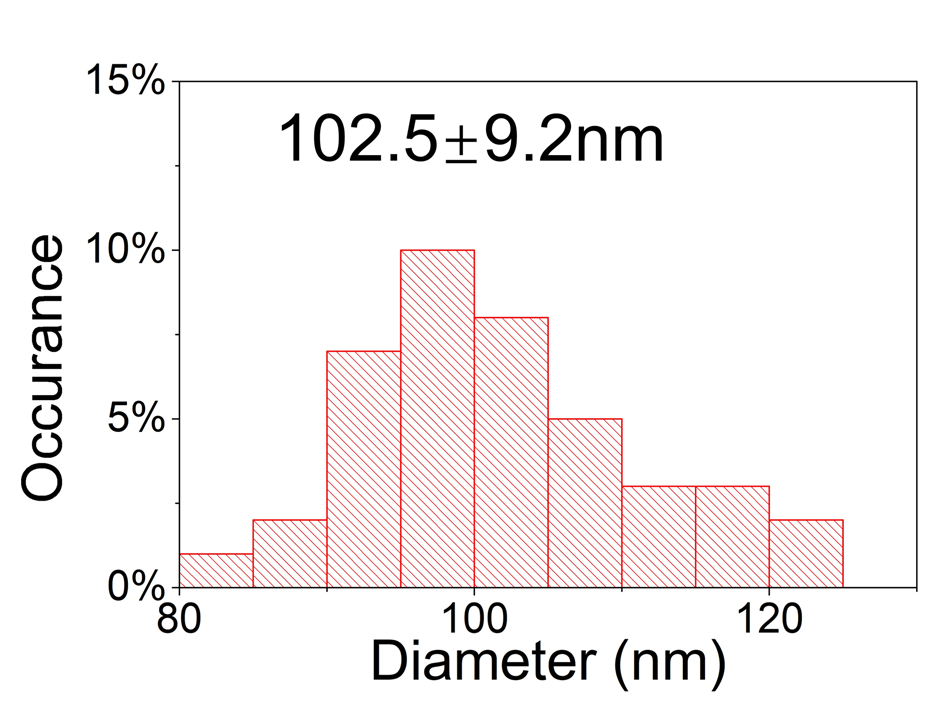

Supplement: Supplementary Figure 1 — Diameters of Nup153 ring measured in FEAST images. [file Image_1.TIF]
